# Some Brief Notes on Theoretical and Experimental Investigations of Intramolecular Hydrogen Bonding

**DOI:** 10.3390/molecules21121657

**Published:** 2016-12-02

**Authors:** Lucjan Sobczyk, Dorota Chudoba, Peter M. Tolstoy, Aleksander Filarowski

**Affiliations:** 1 Faculty of Chemistry, University of Wrocław, 14 F. Joliot-Curie Str., 50-383 Wrocław, Poland; lucjan.sobczyk@chem.uni.wroc.pl; 2 Faculty of Physics, A. Mickiewicz University, Umultowska 85, 61-614 Poznan, Poland; dmn@amu.edu.pl; 3 Frank Laboratory of Neutron Physics, Joint Institute for Nuclear Research, 141980 Dubna, Russia; 4 Center for Magnetic Resonance, St. Petersburg State University, St. Petersburg 198504, Russia; peter.tolstoy@spbu.ru

**Keywords:** intramolecular hydrogen bond, Schiff base, Mannich base, proton sponge, acetophenone, salicylamide, pyridoxal

## Abstract

A review of selected literature data related to intramolecular hydrogen bonding in *ortho*-hydroxyaryl Schiff bases, *ortho*-hydroxyaryl ketones, *ortho*-hydroxyaryl amides, proton sponges and *ortho*-hydroxyaryl Mannich bases is presented. The paper reports on the application of experimental spectroscopic measurements (IR and NMR) and quantum-mechanical calculations for investigations of the proton transfer processes, the potential energy curves, tautomeric equilibrium, aromaticity etc. Finally, the equilibrium between the intra- and inter-molecular hydrogen bonds in amides is discussed.

## 1. Introduction

**Figure 1:**
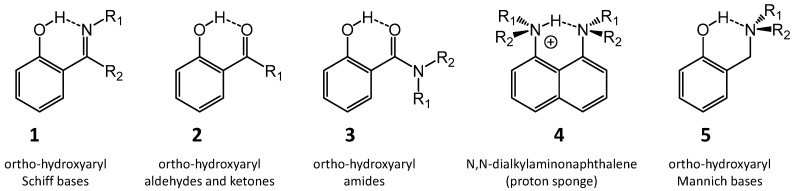
Schematic structures of compounds discussed in this work. R_1_ and R_2_ = hydrogen, aryl and alkyl substitutions.

This paper reports on the studies of the systems with intramolecular hydrogen bonding by experimental (IR, NMR, UV-Vis, X-ray and IINS) and theoretical (DFT, ab initio, AIM) methods. As the primary objects for this short review we have selected Schiff bases, Mannich bases, *ortho*-hydroxyaryl ketones and amides, as well as some proton sponges. A majority of the presented studies covers the phenomena of proton transfer (PT), steric effect, quasi-aromatic hydrogen bonding, aromaticity, tautomeric and conformational equilibria. It should be stressed that the studies of the proton transfer process are of great importance in the description of some enzymatic reactions activated by the hydrogen bonding with a low barrier proton transfer [1,2,3]. The studies of the *ortho*-hydroxyaryl Schiff bases can greatly contribute to the understanding of mechanisms of activity of some drugs as well as support the design of new ones [4,5]. For instance, *ortho*-hydroxyaryl Schiff thiosemicarbazones intercalate between the nitrogen bases in double DNA helix blocking the replication. This feature enables these compounds to be used in the treatment of cancer diseases. There are also substances with *ortho*-hydroxyaldiminecarbazyne structure which coordinate the iron atoms [6,7]. Their presence in the organism regulates the amount of iron in cells, thus preventing the formation of cancer cells. It is important to note that the salicylamides are nonsteroidal anti-inflammatory medicaments. The modelling of the physicochemical features of salicylamides makes it possible to describe the mechanisms of their biological activity. The major types of compounds with intramolecular hydrogen bonds which are discussed in this review (compounds **1**–**5**) are shown in Figure 1.

## 2. Study of Proton Transfer Process in Hydrogen Bridge by Quantum-Mechanical Methods

To study the energetic and geometric properties of intramolecular hydrogen bonds a full spectrum of quantum-mechanical methods could be applied. Concerning the choice of the method and the basis set, usually the recommendations are the same as for other types of H-bonded systems. The level of theory is often limited by the size of the molecule in question. Though MP2 performs admirably [8], the cost-effective DFT methods (usually with B3LYP and PBE functionals) perform quite good as well [9]. The basis set preferably should include diffuse functions. While basis sets such as aug-cc-pVTZ are desired, it seems that 6-311++G(d,p) remains one of the most widely used basis sets up to now. Bader’s “Atoms in Molecules” calculations are often used to estimate the hydrogen bond strength. Calculations are usually performed for isolated molecules, while medium effects are treated by polarized continuum models (PCM) or including one or several solvent molecules explicitly in calculations [10].

### Ortho-Hydroxyaryl Schiff Bases ***1***

**Figure 2:**
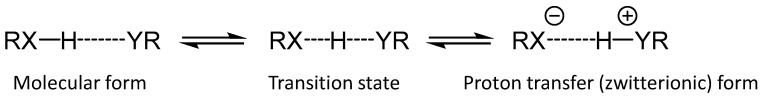
Transition from the molecular form to the proton transfer (zwitterionic) form of the hydrogen-bonded complex.

The papers [11,12,13,14,15,16] present the quantum-mechanical calculations of *ortho*-hydroxyaryl Schiff bases **1** showing the influence of the proton transfer in quasi-aromatic hydrogen bonds on the shape of the potential energy curve as well as on the electron density in critical points of bonds and rings. The authors of [11] elaborated the methodology of the calculations of spectroscopic features and potentials for proton motion in hydrogen bonds. The adiabatic and the non-adiabatic potentials and energy levels for a number of *ortho*-hydroxyaryl ketimines and their deuterated analogues were calculated. From the energy levels, the positions of ν(XH) bands and the H/D isotope effects on them were estimated. Estimation of the anharmonic ν(XH) stretching vibration frequency for the XH bond engaged in hydrogen bond formation is a valuable result because computational programs with standard methods usually do not provide a sufficiently precise description of the ν(XH) band position. In [11,12,15] the calculations of the non-adiabatic potential along the bridging proton transfer coordinate were based on the optimization of all the parameters of the molecule for the gradually elongated XH bond. This approach serves for the description of the changes of structural parameters during the transition from the molecular form to the proton transfer form. It was shown that the proton transfer process leads to the shortening and strengthening of the hydrogen bonding in the first phase of this phenomenon (going from the molecular form to the transition state), and the lengthening in the second phase (going from the transition state to the PT form) (Figure 2).

The paper [11] presents the computational study of the influence of the environment polarity on the potential energy profile for the proton transfer process. The lowering of the energy minimum for the PT form upon increase of the environment polarity for non-adiabatic potential is shown. A similar trend of the adiabatic potential for the molecular form is observed. However, untypical increase of the local minimum of the adiabatic potential for the PT form with the increase of the solvent polarity was also presented.

**Figure 3:**
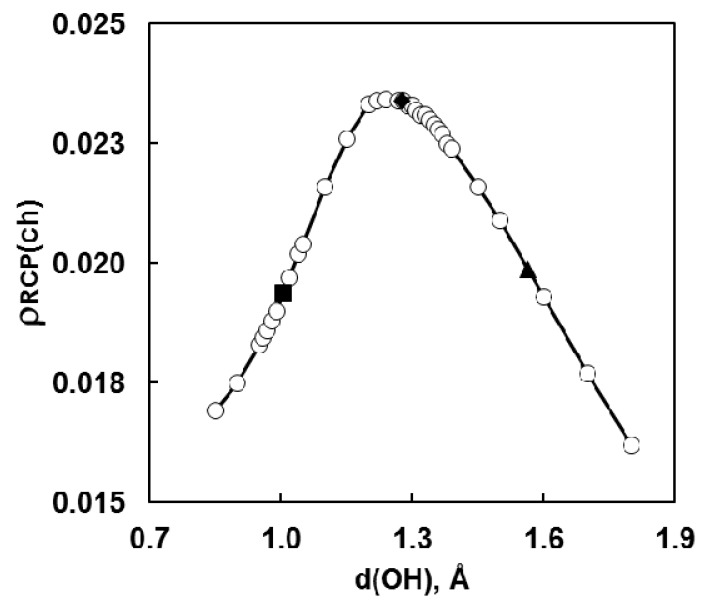
The dependence of the electron densities in the critical point of the quasi-aromatic ring (HO–C=C–C=N) on the length of hydroxyl bond of the *ortho*-hydroxyaryl Schiff base **1** (Figure 1, R_1_ = R_2_ = CH_3_). Dark points correspond to: ■—molecular form, ♦—transition state and ▲—PT form. Reprinted with permission from [12]. Copyright 2008 American Chemical Society.

To describe the changes in the electron densities taking place in a molecule during the proton transfer in hydrogen bridge the “Atoms in Molecules” (AIM) method developed by Bader was applied [12,17,18,19,20,21,22,23]. The changes in the electronic density in the bond critical points (including hydrogen bonds and rings) in the non-adiabatic approach for 2-[(1*E*)-*N*-methyl-ethanimidoyl)]-phenol (**1**, R_1_ = R_2_ = CH_3_; Figure 1) are analysed in [12]. New correlations were obtained and the dependencies presented in the literature [23] were verified. The dependency of the electronic density in the critical point of the chelate chain (O–C=C–C=N) on the length of XH bonding is shown in Figure 3. The electron density at the hydrogen bond critical point goes through a maximum for the transition state (Figure 2), which corresponds to the most linear and shortest hydrogen bridge [24]. It can be seen that the application of the third state is necessary for the description of the tautomeric equilibrium. According to the studies of the potentials this state corresponds to the transition state (Figure 2).

## 3. Spectroscopic Studies of Tautomeric Equilibrium in Intramolecular Hydrogen Bonds

According to a recent IUPAC recommendation, the NMR and IR signatures of a hydrogen bond are the main spectroscopic criteria of its formation [25,26]. As a result, in practice the properties of intramolecular H-bonded systems are often studied experimentally using the values of vibrational frequencies, NMR chemical shifts and coupling constants. As for the majority of H-bonded complexes, in systems discussed here upon formation of the intramolecular hydrogen bond the ν(XH) frequency decreases, while the chemical shifts of the bridging proton increases. Interpretation of these spectroscopic changes in terms of hydrogen bond geometry and energy might be done using previously published correlational dependencies [27,28,29,30,31,32,33,34,35,36]. Despite the typical spectroscopic manifestations of the intramolecular hydrogen bonds, the situation is often complicated by the presence of a tautomeric equilibrium between molecular of proton-transfer form of the complex (except for proton sponges, where both forms are positively charged). The equilibrium constant as well as the characteristics of individual tautomers are temperature-, solvent- and substituent-dependent. Disentangling the overall spectroscopic patterns requires a careful analysis and usage of other spectroscopic markers, such as, i.e., UV-Vis band positions and intensities, C=O stretching frequencies, ^13^C-, ^15^N-NMR chemical shifts, spin-spin coupling constants and H/D isotope effects on all of the abovementioned parameters.

### 3.1. Ortho-Hydroxyaryl Schiff Bases ***1***

Spectroscopic studies including IR and Raman spectroscopic data as well as incoherent inelastic neutron scattering (IINS) data were presented in the literature [37,38,39,40,41,42,43,44]. These studies were based on a series of spectra recorded under the argon matrix condition, in the gas phase and the solid state. A detailed analysis of vibration spectra was also carried out by quantum-mechanical calculations. The effects of deuteration on band positions and intensities were investigated for the cases of deuteration in the hydrogen bridges and in the methyl group of imine fragments (N=C–CH_3_) in *ortho*-hydroxyaryl ketimines (**1**, R_2_ = CH_3_; Figure 1). The position of the ν(C–O) band turns out to be sensitive to the deuteration in the hydrogen bridge for the molecular form, while for the PT form the deuteration influences the ν(C=N) band. This effect makes it possible to estimate the proton position in the hydrogen bridge.

**Figure 4:**
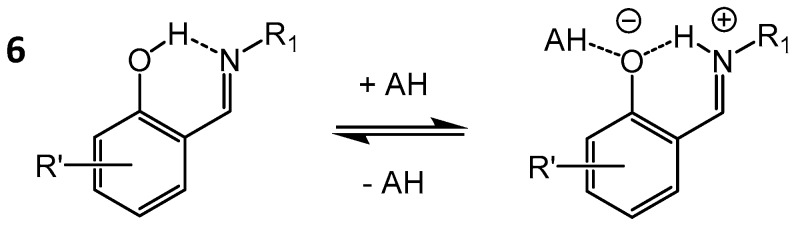
Schematic structure of *ortho*-hydroxyaryl Schiff bases, studied by NMR in [46].

In [45,46,47] various solutions of partially deuterated and ^15^N-labelled *ortho*-hydroxyaryl Schiff bases **6** (Figure 4) were studied by ^1^H- and ^15^N-NMR spectroscopy, revealing the presence of intramolecular proton tautomerism between molecular (enol-imine) and zwitterionic (keto-amine) forms.

The analysis of substituent- and temperature- dependent ^1^H and ^15^N chemical shifts has revealed that upon the increase of the solvent polarity two effects are present: (i) shift of the tautomeric equilibrium towards the zwitterionic structures and (ii) changes in the hydrogen bond geometry in each of the tautomers. Namely, the hydrogen bond in the molecular form becomes stronger (symmetrization of the bridging proton position) while that in the zwitterionic tautomer becomes weaker (asymmetrization). A similar effect was later observed for other strongly hydrogen bonded systems and it was ascribed to the non-specific [48,49] or in some cases to specific [50] solvent-solute interactions. For *ortho*-hydroxyaryl Schiff bases **6** (Figure 4) the formation of additional hydrogen bonds to the phenol/phenolate oxygen atom by one or several alcohol molecules was shown to induce the proton transfer and stabilization of the keto-amine form [45,46].

**Figure 5:**
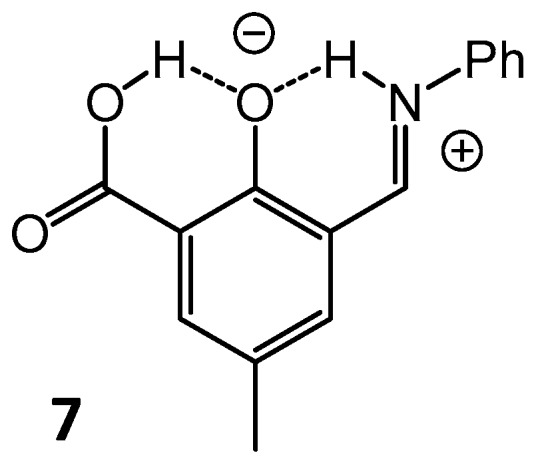
Schematic structure of *ortho*-hydroxyaryl Schiff base **7**, studied by NMR in [51].

In [51] the effect of an additional covalently linked carboxylic group on the intramolecular proton tautomerism in 2-carboxy-4-methyl-6-[(*E*)-(phenyliminio)methyl]phenolate (*ortho*-hydroxyaryl Schiff base, **7**, Figure 5) has been studied. It was shown that in a CDF_3_/CDF_2_Cl solution the additional COOH group forms an OHO hydrogen bond with the phenolic oxygen atom, which “pushes” the proton in the neighboring OHN bonds towards the nitrogen atom. As a result, **7** exists almost exclusively in a proton transfer form OH···O^−^···HN^+^, in which two proton donors form coupled hydrogen bonds and compete for the central phenolate oxygen acceptor.

**Figure 6:**
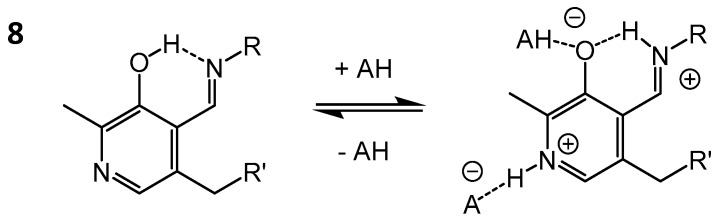
Schematic structure of *ortho*-hydroxy Schiff bases **8**, formed by PLP, studied by NMR ([59] and references cited therein).

An interesting case of proton tautomerism and hydrogen bond coupling is represented by pyridoxal 5′-phosphate (PLP), which forms *ortho*-hydroxyaryl Schiff bases **8** (Figure 6) as a cofactor of many enzymes and also during the course of its catalytic cycle (so called “internal” and “external” aldimines, respectively) [52].

Protonation states, hydrogen bond geometries and hydrogen bond coupling in PLP has been studied recently by NMR in aqueous solution [53,54], in aprotic medium [55], in solid state [56,57] and also in hydrophobic pockets of proteins (alanine racemase, AlaR, and aspartate aminotransferase, AspAT) [58,59]. It was demonstrated that the proton position in the intramolecular OHN bond is determined to a large extent by the additional bonds formed with the phenolic oxygen or pyridine nitrogen atoms of PLP. In both cases, the formation of additional hydrogen bonds shifts the equilibrium towards the catalytically active zwitterionic ketoamine form. For example, in AspAT the pyridine nitrogen atom of PLP is fully protonated by the COOH group of the neighboring aspartic acid side chain [58].

### 3.2. Ortho-Hydroxyaryl Aldehydes and Ketones ***2***

*Ortho*-hydroxyaryl aldehydes and ketones are structurally close to *ortho*-hydroxyaryl Schiff bases, but they do not display proton transfer in the ground state [60,61,62]. The studies of two derivatives of *ortho*-hydroxyacetophenone (5-cyano-2-hydroxyacetophenone and 2-hydroxy-4-methoxy-5-nitro-acetophenone) [62] which possess markedly strong acidity of the hydroxyl group failed to show the existence of the PT form in the ground state. Moreover, the investigation of fluorescent spectra recorded in solution and in the solid state show the prevailing of the PT form in the excited state for the *ortho*-hydroxyacyl aromatic compounds [63,64,65].

### 3.3. Ortho-Hydroxyaryl Mannich Bases ***5***

The studies of adiabatic potentials for the proton motion in 3,5,6-trimethyl-2-(*N*,*N*-dimethylaminomethyl)phenol (**5**, R_1_ = R_2_ = CH_3_; Figure 1) and the description of infrared spectra by means of Car-Parrinello molecular dynamics (CPMD) is presented in [51]. The strengthening of the intramolecular hydrogen bond in this compound due to the steric effect of the methyl substituents in the phenyl ring was earlier stated and proved experimentally [66]. The aim of paper [67] was the verification of the conformity of the applied computational methods with the description of the intramolecular hydrogen bonding. The presented calculations for solution and solid states of 3,5,6-trimethyl-2-(*N*,*N*-dimethylamine-methyl)phenol by CPMD has allowed the authors to describe both spectral and structural parameters. The potential energy curves and corresponding vibrational energy levels estimated at B3LYP/cc-pVDZ and MP2/cc-pVDZ levels of theory for the adiabatic proton movement also made it possible to calculate the position of the ν(OH) stretching band. It was stated that the resulting calculated frequency is in a satisfactory agreement with experimental data. It should be underlined that CPMD method allows to study chemical reactions dynamically and to compute free energy profiles via thermodynamic integration of fluctuations and, therefore, to calculate entropy [68]. In [69,70] the potential energy curves of proton transfer in intramolecular hydrogen bond in *ortho*-hydroxyaryl Schiff base [69] and acetic acid dimer [70] were studied, and the consistency of CPMD method was shown.

**Figure 7:**
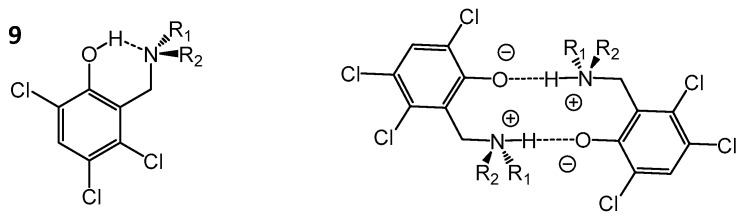
Schematic structure of *ortho*-hydroxyaryl Mannich base **9** (**left**) and its zwitterionic cyclic dimer (**right**), studied by NMR in [74].

In liquid-state NMR spectra of *ortho*-hydroxyaryl Mannich bases the intramolecular OHN hydrogen bond manifests itself by a low-field shift of the bridging proton signal [71,72,73,74] and in some cases by the inversion of the sign of H/D isotope effects on ^13^C-NMR chemical shifts upon temperature decrease [75]. The latter is associated with the shift of the proton equilibrium from the molecular OH···N form to the zwitterionic O^−^···HN^+^ form. In [74] compound **9** (Figure 7) was studied by a combination of NMR and UV/Vis spectroscopy showing that in a polar aprotic medium such as a mixture of Freon gases CDF_3_/CDF_2_Cl the molecule exists in a molecular OH···N form, which changes to the zwitterionic O^−^···HN^+^ form upon the formation of cyclic dimers (shown in Figure 7, right). The existence of the zwitterionic forms for monomers of compound **9** was not confirmed.

### 3.4. Dimethylaminonaphthalene (***4***)

**Figure 8:**
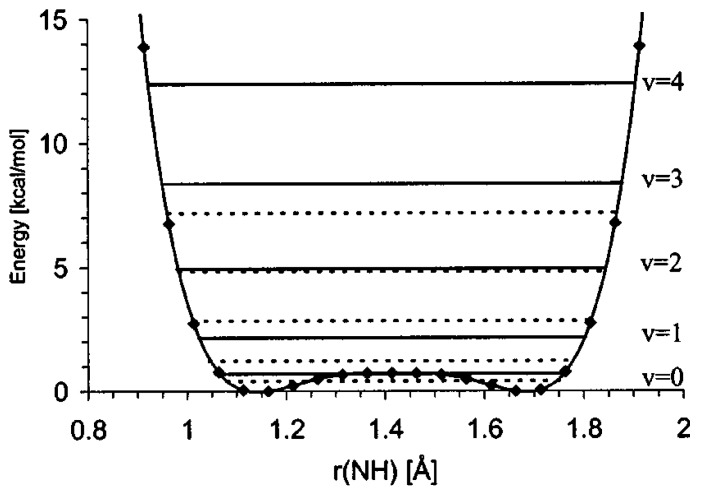
The potential energy curve for protonated 2,7-dibromo-1,8-bis(dimethylamino)naphthalene obtained by MP2/6-31+G(d,p) level of theory. Reprinted with permission from [79]. Copyright 2008 AIP Publishing.

Dimethylaminonaphthalene, so-called proton sponge, is the subject of intensive studies [76,77,78,79,80,81,82,83,84,85,86,87,88,89]. In [76,77,78,79,80] the methods of synthesis, the analysis of the structural characteristics and the results of ab initio (MP2/6-31+G(d,p)) calculations for a series of dimethylaminonaphthalene derivatives are presented. It is noteworthy that the results of quantum-mechanical calculations and the interpretation of the NMR spectra of proton sponges with a strong buttressing effect coincide. For example, the calculated potential for the proton transfer for 2,7-dibromo-1,8-bis(dimethylamino)naphthalene is symmetric and possesses a low energy barrier (Figure 8) [79]. This result points out the delocalization of the proton in the hydrogen bridge which is in accordance with the record ^1^H-NMR chemical shift δ(^1^H) = 21–22 ppm [81,82] and a very high isotopic spectroscopic ratio, *ISR* = ν(NHN)/ν(NDN) = 1.8 [83].

**Figure 9:**
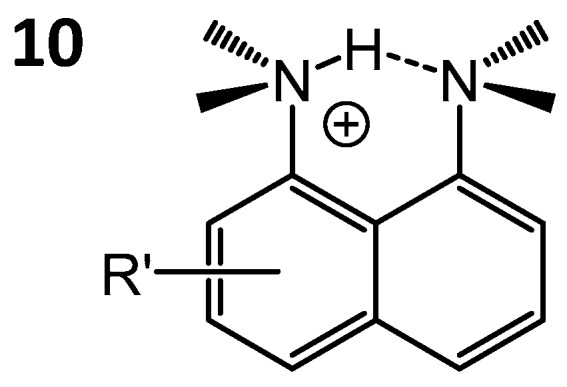
Schematic structure of protonated proton sponge **10**, studied by NMR in [86].

The NMR studies of hydrogen bonding in proton sponges of type **10** (Figure 9) are mostly based on the interpretation of ^1^H-, ^15^N-NMR chemical shifts, as well as ^1^*J*_NH_, ^1h^*J*_NH_ and ^2h^*J*_NN_ spin-spin coupling constants (the latter can be detected directly [84] or indirectly [68]) and H/D isotope effects on them.

In [86] the (NHN)^+^ hydrogen bond geometry and symmetry was studied depending on the solvent, counter-anion and temperature used (solvents: CD_3_CN, CD_2_Cl_2_, toluene-D_8_ and CDF_3_/CDF_2_Cl mixture; counter-anions: ClO_4_^−^, trifluoroacetate (TFA^−^), tetrakis[3,5-bis(trifluoro- methyl)phenyl]borate (BARF^−^)). It was shown that the positive charge in the (NHN)^+^ bond is well localized, indicating the presence of a degenerate tautomeric equilibrium between NH^+^···N and N···^+^NH forms (asymmetric proton position was also detected in some proton sponges by dipolar solid-state NMR [87]). Lowering the temperature increases the symmetry of the hydrogen bond, as evidenced by the changes in NMR parameters; this phenomenon was attributed to the increase of the polar solvent ordering around the charged hydrogen bridge. It was also noticed that small counter-anions tend to perturb the hydrogen bond symmetry (presumably, due to the asymmetrical placement). In contrast, usage of large counter-anions with strongly delocalized negative charge leads to hydrogen bond symmetrization. A similar effect was studied later for other NHN-bonded systems [88,89].

## 4. Potential for Proton Transfer and Tautomeric Equilibrium

**Figure 10:**
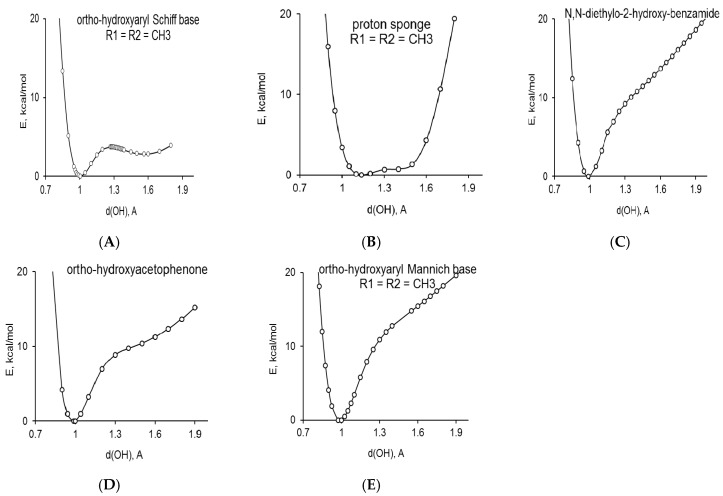
The non-adiabatic potential curves for: *ortho*-hydroxyaryl Schiff base (**1**; R_1_ = R_2_ = CH_3_, Figure 1) (**A**); 1,8-bis(dimethylamino)-2,7-bis(trimethylsilyl)naphthalene (derivative of proton sponge) (**4**; R_1_ = R_2_ = CH_3_; Figure 1) (**B**); 2-hydroxy-*N*,*N*-diethylbenzamide (**3**; R_1_ = R_2_ = C_2_H_5_; Figure 1) (**C**); *ortho*-hydroxyacetophenone (**2**; R_1_ = CH_3_; Figure 1) (**D**) and *ortho*-hydroxyaryl Mannich base (**5**; R_1_ = R_2_ = CH_3_; Figure 1) (**E**).

The studies of the non-adiabatic potential for the proton transfer in *ortho*-hydroxyaryl Schiff bases [11,90], *ortho*-hydroxyaryl ketones [91], *ortho*-hydroxyaryl Mannich bases [66,92,93], dimethylaminonaphthalene [76,77,78,79,80] and *ortho*-hydroxyaryl amides [94] confirm the presence of the second local minimum on the potential curve for some *ortho*-hydroxyaryl Schiff bases and proton sponges (Figure 10), which allows one to observe tautomeric equilibrium. The potential energy curves do not exhibit a second a local minimum for *ortho*-hydroxyaryl amides, *ortho*-hydroxyaryl ketones, *ortho*-hydroxyaryl Mannich bases and, therefore, the experimental observation of the stable PT tautomeric form is not possible.

## 5. Proton Transfer Process and Aromaticity

### Ortho-Hydroxyaryl Schiff Bases ***1***

**Figure 11:**
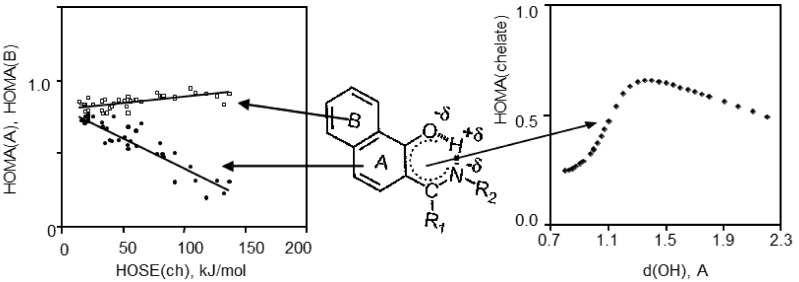
Left side: correlations between HOMA(A), HOMA(B) and (HOSE(ch)) aromaticity indexes calculated on the X-ray diffraction data. Reprinted with permission from [100]. Copyright 2008 John Wiley & Sons, Ltd. Right side: dependency of the aromaticity of chelate chain (HOMA(ch)) depending on the OH bond length calculated on MP2/6-31+(d,p) data. R_1_ and R_2_ substitutions are hydrogen, alkyl and aryl substitutions.

**Figure 12:**
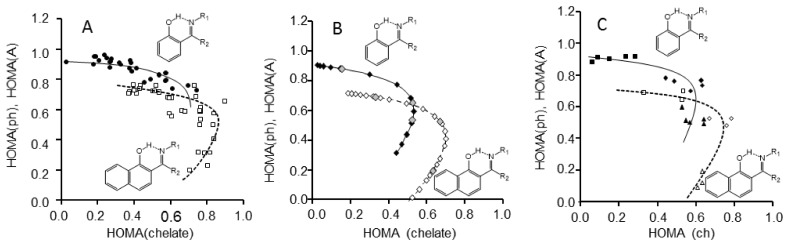
Dependencies of the HOMA aromaticity index of phenyl ring (HOMA(ph)) (solid line) and naphthalene (HOMA(A)) (dotted line) on the changes of aromaticity of chelate chain (HOMA(ch)) obtained from the experimental data (**A**); calculated with non-adiabatic approach for 2-[(1*E*)-*N*-methylethanimidoyl]phenol (R_1_ = R_2_ = CH_3_) and 2-[(1*E*)-1-(methyliminio)ethyl]-naphthalen-1-olate (R_1_ = R_2_ = CH_3_) molecules (**B**) and for fully optimised tautomeric form (**C**).

The formation of the intramolecular quasi-aromatic hydrogen bonding via π-electronic delocalization in the systems with the alternated double bonds (so-called chelate chain) stabilizes a flat conformation of a molecule and enforces hydrogen bonding. This effect is known as Resonance Assisted Hydrogen Bond (RAHB) [95]. From the point of view of the acid-base interactions, such strengthening of hydrogen bonding is caused by the mutual increase of basicity and acidity due to the +M and −M mesomeric effects via the chelate chain [96,97]. Recently, some literature sources have put in question the abovementioned point of view [98]. Yanez et al. [98] demonstrate that π-electronic conjugation does not strengthen hydrogen bonding. Obviously, the studies of aromaticity are very important for answering the question about the strengthening of hydrogen bonding in quasi-aromatic systems. References [99,100,101,102,103] dwell on the description of the aromaticity changes in systems with intramolecular hydrogen bonding depending on the state of tautomeric equilibrium. The papers [104,105,106,107,108] demonstrate that the parameters of aromaticity HOMA and HOSE [104] are reliable indicators in the estimation of tautomeric equilibrium. Based on X-ray diffraction data the correlations between the HOMA and HOSE aromaticity indexes and the XH bond length were obtained [100]. The phenyl and naphthalene derivatives of Schiff bases show the existence of aromaticity equilibrium, which reflects the decrease of aromaticity of the A ring with a little increase of aromaticity of the B ring under the proton transfer process (HOMA(A,B) = *f*(HOSE(ch)), Figure 11). It was stated that the change from the molecular form to the transition state triggers the increase of aromaticity of the chelate chain, and on the contrary, the change from the transition state to the PT form evokes the decrease of aromaticity (HOMA(ch) = f(d(OH)), Figure 11). It was also shown that the changes of aromaticity of the chelate chain (HOMA(ch)) depend in a non-linear way on the changes of aromaticity of the phenyl ring (or naphthalene ring A) under the proton transfer (HOMA(ph) = *f*(HOMA(ch)), Figure 12).

An interesting fact is that the calculated results obtained by the non-adiabatic approach give a precise description of the aromaticity changes.

## 6. Equilibrium between Different Intra-/Intra-Molecular Hydrogen Bonding and Intra-/Inter-Molecular Hydrogen Bonding

### 6.1. Ortho-Hydroxyaryl Aldehydes and Ketones ***2***

**Figure 13:**
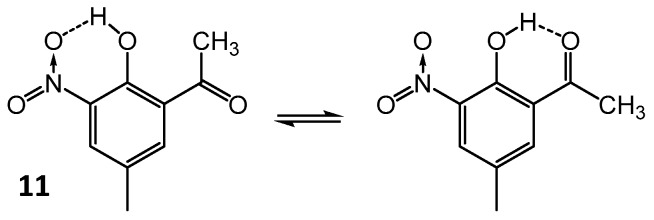
Scheme of the hydroxyl group isomerization in 5-methyl-3-nitro-2-hydroxyacetophenone (**11**).

The phenomenon of competition between two hydrogen bonds within one molecule is an interesting point to consider [109,110]. The competitive equilibrium between the O–H···O_2_N– and O–H···O=C hydrogen bonds in 5-methyl-3-nitro-2-hydroxyacetophenone (**11**) (Figure 13) by infrared spectroscopy and quantum-mechanical calculations is studied in [91,110].

**Figure 14:**
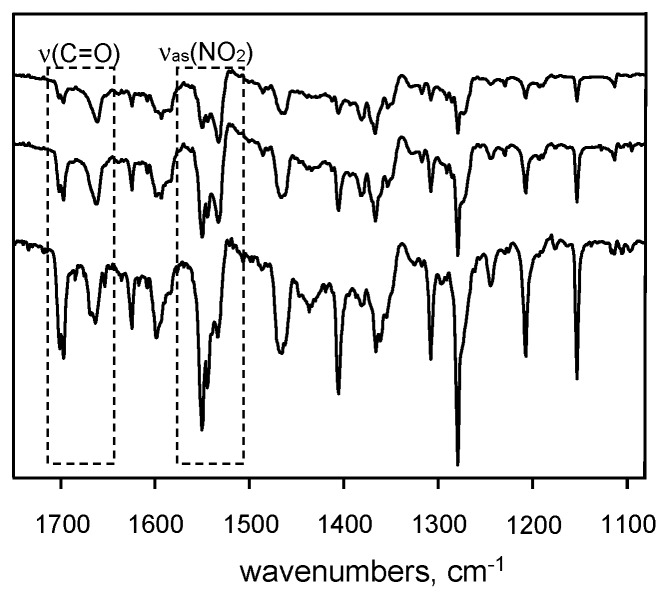
Spectrum of 5-methyl-3-nitro-2-hydroxyacetophenone (**11**) in argon matrix condition in the function of sublimation. Reprinted with permission from [91]. Copyright 2008 Elsevier.

The infrared spectra recorded under argon matrix condition as a function of the sublimation temperature revealed significant changes in the intensity of the ν(C=O) and ν(NO_2_) stretching vibration bands (Figure 14). The result was used to attribute particular bands to two isomers of the compound.

### 6.2. Ortho-Hydroxyaryl Amides (***3***)

An amide bond in proteins plays an essential role in the functioning of every living organism, therefore, the description of the conformation of such systems is very important. Papers [94,111] showed the importance of complex studies of *ortho*-hydroxyaryl amides. The ^1^H and ^13^C-NMR spectra of *ortho*-hydroxyaryl amides studied as a function of temperature demonstrate the presence of a dynamic effect evoked by the non-equivalent orientation of dimethylamine, diethylamine, morpholine and pyrrolidine groups [94,111].

**Figure 15:**
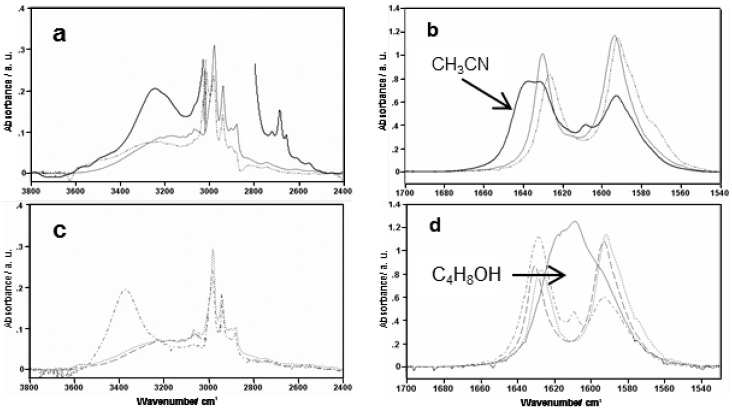
Infrared spectra of 2-hydroxy-*N*,*N*-diethylbenzamide (**3**, R_1_ = R_2_ = C_2_H_5_; Figure 1) in the region of the ν(OH) (left side) and ν(C=O) (right side) modes for 2-hydroxy-*N*,*N*-diethylbenzamide: (**a**,**b**) solid line—CCl_4_, .. line—CHCl_3_, bold line—C_4_H_8_O; (**c**) - - - line—C_4_H_9_Cl, .. line—CH_2_Cl_2_, -.-.- line—CH_3_CN; (**d**) - - - line—C_4_H_9_Cl, .. line—CH_2_Cl_2_, -.-.- line—CH_3_CN, solid line—C_4_H_9_OH. Reprinted with permission from [94]. Copyright 2008 John Wiley & Sons, Ltd.

**Figure 16:**
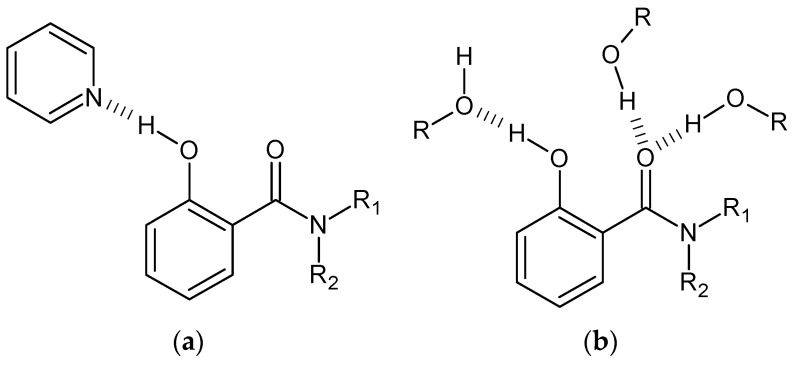
Complexes of 2-hydroxy-*N*,*N*-diethylbenzamide (**3**, R_1_ = R_2_ = C_2_H_5_; Figure 1) with pyridine (**a**) and alcohol (**b**) (R = alkyl group). Reprinted with permission from [94]. Copyright 2008 John Wiley & Sons, Ltd.

The infrared spectra (Figure 15) recorded as a function of temperature in weakly-polar, proton-donating and proton-accepting solvents indicate the existence of equilibrium between intramolecular and intermolecular hydrogen bonds [94]. It should be underlined that an easy disruption of a rather strong intramolecular hydrogen bond observed in 2-hydroxy-*N*-methylbenzamide [112], salicylamide [113] and 4-chloro-2-hydroxybenzamide [114] (d(OO) = 2.53–2.47 Å) is an outcome of a steric effect of the ethyl groups in 2-hydroxy-*N*,*N*-dialkylbenzamides and a weakened conjugation between the phenyl and amide moieties due to competitive resonance [94,111]. Further investigations of infrared spectra revealed the shift of the ν(C=O) bands depending on the solvent polarity (Figure 15). In the case of proton-accepting solvent, we observe the disruption of intramolecular hydrogen bonding and the formation of the complex with the solvent molecules, which shifts the ν(C=O) band towards higher wavenumbers with respect to that in weakly-polar solvent. This fact is explained by the disruption of hydrogen bonding (Figure 16) and the strengthening of the force constant of this bond. The shift of the ν(C=O) band in the case of the amphoteric solvent is drastically different (the shift towards lower wavenumbers) from the proton-acceptor solvent. Such effect is conditioned by the formation of intermolecular hydrogen bonding between the carbonyl group and the solvent molecules, which weakens the carbonyl bond and decreases force constant.

**Figure 17:**
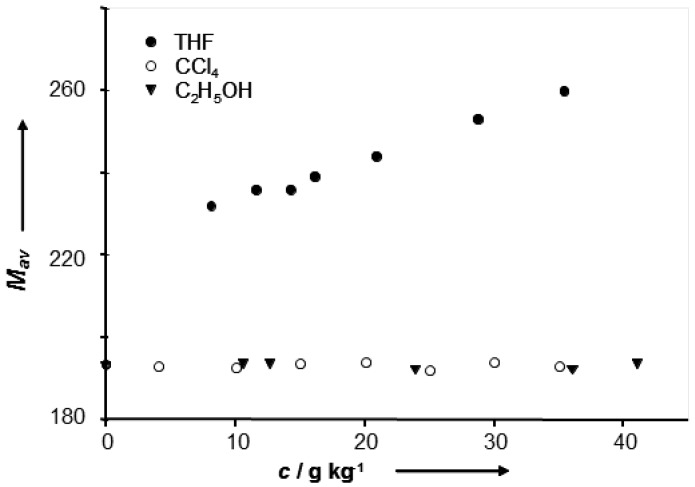
Dependence of average molecular weight (M_av_) on concentration. Reprinted with permission from [94]. Copyright 2008 John Wiley & Sons, Ltd.

The measurements of an average molecular mass (Figure 17) also exhibit ambiguous influence of amphoteric and proton-acceptor solvents. These results point out the presence of self-association but only in proton-accepting solvents. Different behaviour of a compound in amphoteric and proton-accepting solvents is due to the blocking of a basic center of the molecule, which prevents the formation of the complex (Figure 16). This hypothesis is supported by the results obtained from the infrared measurements.

## 7. Short Summary

Hydrogen bonding is one of the main concepts in contemporary chemistry [63,95,104,108,115,116,117,118,119,120,121,122,123,124,125,126,127] and its study is important for the development of the discipline. Intramolecular hydrogen bonds in *ortho*-hydroxyaryl Schiff and Mannich bases, in *ortho*-hydroxyaryl ketones and amides, in proton sponges and related compounds could be considered as classical objects for the investigation. Though the presented short review does not cover the topic in its entirety, we have tried to outline the main current research directions. On the one hand, the geometric and spectroscopic characteristics of the abovementioned intramolecular hydrogen bonds are typical for H-bonds of medium strength (for an XHY bond it is short XY contact, XH elongation, ordinary directionality pattern, ν(XH) frequency decrease, bridging proton deshielding etc.). On the other hand, there are enough specific properties, which make the study of such bonds a topic in its own right. Perhaps the most prominent feature is a possibility of proton delocalization due to the prototropic equilibrium between molecular and zwitterionic forms, possible in some systems. The tautomeric equilibrium is temperature-, solvent- and substituent dependent; it is also strongly influenced by additional intermolecular hydrogen bonds formed by the molecule (with or without breaking the intramolecular bond). Other sources of proton delocalization are the formation of stronger intramolecular hydrogen bonds with broader proton potentials and thermally fluctuating medium effect, which are influencing H-bond geometry. The equilibrium constant of the tautomeric equilibrium changes together with the intrinsic geometric and spectroscopic properties of individual tautomers, which complicates the analysis of the experimental data. Here, quantum-chemical computations are often providing the necessary insight via adiabatic and non-adiabatic PES calculations. Another interesting feature of intramolecular H-bond discussed in this short review is the stabilization provided by the π-electron delocalization in the chelate chain, which gives the name Resonance-Assisted Hydrogen Bonds (RAHB).
